# Sperm competition and fertilization mode in fishes

**DOI:** 10.1098/rstb.2020.0074

**Published:** 2020-10-19

**Authors:** John L. Fitzpatrick

**Affiliations:** Department of Zoology/Ethology, Stockholm University, Svante Arrhenius väg 18B, Stockholm 10691, Sweden

**Keywords:** promiscuity, sperm design, extra-pair paternity, sexual selection

## Abstract

Sperm competition is a powerful selective force that has shaped sexual traits throughout animal evolution. Yet, how fertilization mode (i.e. external versus internal fertilization) influences the scope and potential for sperm competition to act on ejaculates remains unclear. Here, I examine how fertilization mode shapes ejaculatory responses to sperm competition in fishes, a diverse group that constitute the majority of vertebrate biological diversity. Fishes are an ideal group for this examination because they exhibit a wide range of reproductive behaviours and an unparalleled number of transitions in fertilization mode compared to any other vertebrate group. Drawing on data from cartilaginous and bony fishes, I first show that rates of multiple paternity are higher in internally than externally fertilizing fishes, contrary to the prevailing expectation. I then summarize how sperm competition acts on sperm number and quality in internally and externally fertilizing fishes, highlighting where theoretical predictions differ between these groups. Differences in how ejaculates respond to sperm competition between fertilization modes are most apparent when considering sperm size and swimming performance. Clarifying how fertilization mode influences evolutionary responses in ejaculates will inform our understanding of ejaculate evolution across the animal tree of life.

This article is part of the theme issue ‘Fifty years of sperm competition’.

## 1. Introduction

Sperm competition, the competition between sperm from rival males to fertilize ova [1], is a widespread evolutionary force that has played a major role in shaping animal reproductive anatomy, physiology and behaviour throughout their evolution [1–4]. Ancestral gamete competition between proto-male and proto-female gametes is hypothesized to be responsible for the evolution of anisogamy, with differences in gamete sizes being maintained by sperm competition [3,4]. Anisogamy set the stage for a series of transitions in sexual strategies (a ‘sexual cascade’) that helped generate the extraordinary diversity in sexual traits observed in animals [3]. The strength of sperm competition is generally expected to weaken throughout this sexual cascade (as precopulatory sexual selection intensifies), with transitions from external to internal fertilization altering how selection acts on ejaculates [3,4].

Fish exhibit extraordinary diversity in their mating behaviours, both within and between species, making them an excellent model for studying sperm competition [5–7]. However, this behavioural diversity is nested within broader diversity in sexual strategies. A fundamental difference in reproductive biology among fishes is in fertilization mode, classified broadly as fertilization that occurs either outside (i.e. external fertilization) or within (i.e. internal fertilization) the female's reproductive tract. At least 12 independent evolutionary transitions from external to internal fertilization occur in fishes [8], a number that is unparalleled compared to any other vertebrate group [8,9]. Thus, fishes offer an exceptional opportunity to study how variation in fertilization mode influences how selection acts on ejaculates [10].

Yet how fertilization mode influences sperm competition levels and selection on ejaculates remains unclear. External fertilizers are assumed to have less control over paternity, as external release of gametes allows for group spawning and sneaking behaviours [5,11]. Sperm competition levels are, therefore, generally thought to be higher in externally fertilizing species compared with internal fertilizers [5,11]. Whether this assumption is valid is unclear. Both internally and externally fertilizing fishes show wide variation in mating behaviours [5], which may erode differences in sperm competition levels between fertilization modes [3], and sperm storage by females can intensify sperm competition levels in internal fertilizers [4]. Fertilization mode also introduces stark differences in the environments where ejaculates operate, which can influence responses to sperm competition. For example, ejaculates from external fertilizers are likely subject to greater dilution and stochastic environmental effects (e.g. [12]) and typically have reduced intervals between gamete release and fertilization compared with internal fertilizers. Such differences in fertilization environment have the potential to alter how sperm competition acts on sperm number, size and longevity ([13–16], table 1). Despite these differences, comparisons of how sperm competition and selection on ejaculates are influenced by fertilization mode are rare [10,21].

**Table 1.** Sperm competition, fertilization mode and ejaculates. I summarize predictions from sperm competition models for (*a*) sperm number, including testes size and sperm allocation under both risk and intensity models; and (*b*) sperm quality, focusing on sperm size, swimming speed and longevity. For each ejaculate trait, I summarize the main predictions from sperm competition models, discuss how fertilization mode can influence these predictions, and comment on general empirical patterns across animals.

ejaculate trait	predictions, fertilization mode and empirical patterns
(*a*) sperm number	
testes size (relative testes size)	males are expected to increase their relative (i.e. correcting for body size) investment in testes size in response to increasing sperm competition risk and intensity both within and across species [17]. This prediction is sensitive to the strength of raffle loading and costs of acquiring a mate [17], but does not differ between external and internal fertilizers. However, a recent meta-analysis demonstrates that effect size estimates of the relationship between testes size (correcting for body size) and sperm competition are lower in externally than internally fertilizing animals [18]. Sperm dilution/sperm limitation effects may influence evolutionary responses in sperm number in external fertilizers compared to internal fertilizers
sperm allocation	
sperm competition risk models	sperm allocation is expected to increase with sperm competition risk, but this prediction is sensitive to a wide range of moderators [17]. Unless moderating factors consistently vary between fertilization modes, predictions are similar for external and internal fertilizers. A recent meta-analysis found that external and internal fertilizers allocate more sperm when sperm competition risk is high [19]
sperm competition intensity models	sperm allocation is predicted to progressively decrease as sperm competition intensity increases above two competitors [17]. Predictors vary depending on a wide range of moderators [17]. In particular, model predictions are influenced by the amount of information available to competing males, which may differ based on fertilization mode. For example, external fertilizers may be better able to assess the number of competitors present when allocating sperm, particularly compared with sequentially mating internal fertilizers. Effect size estimates obtained from studies testing the intensity model are in opposite directions for external (negative) and internal (positive) fertilizers, although neither differed from zero [19]
(*b*) sperm quality	
sperm size and swimming speed, and longevity	existing models focus on sperm size, drawing distinctions between fertilization where sperm and eggs are shed simultaneously (i.e. external fertilization) and where sperm survival is modelled after release (i.e. internal fertilization). Predictions depend on the relationships between sperm size and swimming speed (often assumed to be positive) and sperm size/speed and longevity (often assumed to be negative) [14]. Slower but longer-lived sperm are predicted with increasing sperm competition in internal fertilizers [15]. Longer and faster-swimming sperm (that are short lived) are expected to be favoured in external fertilizers where sperm must rapidly reach and fertilize eggs before those of rivals, although predictions are sensitive to model assumptions [14]. The relationship between sperm size and swimming speed are more apparent in external than internal fertilizers [20], suggesting a clearer link with model assumptions in this group

Here, I examine how fertilization mode shapes the scope and potential for sperm competition in fishes, drawing on examples from both cartilaginous (class Chondrichthyes) and bony (confined to teleost, infraclass Teleostei) fishes. I first use genetic estimates of multiple paternity rates to test the assumption that sperm competition is higher in externally fertilizing fishes. I then examine whether and how predictions from sperm competition theory differ based on fertilization mode, followed by a summary of how sperm competition influences the evolution of sperm number and quality (i.e. sperm morphology, longevity, swimming speed and viability [16]) in externally and internally fertilizing species separately. Finally, I identify commonalities and differences in patterns of selection on ejaculates between fertilization modes and highlight fruitful avenues for future consideration.

## 2. Multiple paternity and fertilization mode in fishes

Multiple paternity, which offers a minimum estimate of female multiple mating (and thus sperm competition level), has been studied extensively in natural populations of fishes [21]. In fishes, multiple paternity is typically quantified as the proportion of nests/clutches where offspring were sired by greater than one males (in external fertilizers) or the proportions of broods/litters where greater than one males sire progeny (in internal fertilizers). These measures of multiple paternity rates, which are most common in the literature, serve as a proxy for sperm competition risk (i.e. the probability of competing with another ejaculate [17]) and are tightly correlated with alternative metrics of multiple paternity (e.g. mean number of sires per brood, a proxy for sperm competition intensity [22]).

**Figure 1.**
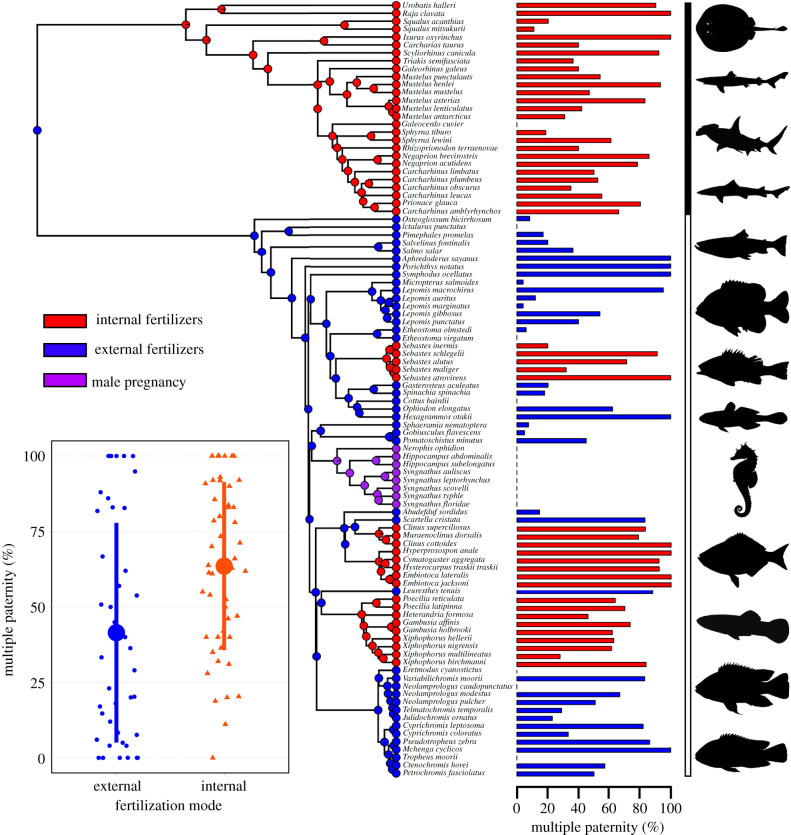
Rates of multiple paternity and fertilization mode in 98 species of fishes. Phylogenetic distribution showing the ancestral state reconstruction of fertilization mode for internally (red) and externally (blue) fertilizing fishes, and seahorses and pipefish species that exhibit male pregnancy (purple). Rates of multiple paternity across fishes are presented in the bar plots. The black bar on the right side of the plot indicates internally fertilizing sharks and rays (Elasmobranchs) while the white bar indicates internally and externally fertilizing bony fishes. Mean (±standard deviation) rates of multiple paternity for internal and external fertilizers are summarized in the inset plot, with raw data presented as jittered points. Species with male pregnancy are excluded from the comparison of fertilization modes as these species have vast differences in their reproductive biology that prevent a meaningful comparison. Fish silhouettes were obtained from http://www.phylopic.org and are licenced for use in the Public Domain without copyright.

I collated 25 years of data on genetic estimates of paternity in fishes and examined the effect of fertilization mode on rates of multiple paternity in a phylogenetic framework (figure 1; electronic supplementary material). This dataset included external (*n* = 41) and internal (*n* = 49) fertilizers, as well as species with male pregnancy (e.g. pipefish and seahorses, *n* = 8), the latter of which were treated separately given their unusual reproductive strategy. Rates of multiple paternity are variable in both internal and externally fertilizing fishes (figure 1). Contrary to expectations, however, rates of multiple paternity were significantly higher in internally fertilizing fishes (PGLS regression, *λ* < 0.01, *n* = 90, *t* = 3.84, *p* < 0.001), with mean multiple paternity rates 22% higher in internal than external fertilizers (figure 1, inset). Whether this result is robust across fishes, in general, is uncertain. While greater than 95% of fishes are external fertilizers, the available data comprised more internal than external fertilizers. This sampling bias is due in part to the relative ease of collecting broods/litters from internal fertilizers. The external fertilizers included in the analysis are skewed towards species with male nest defence or biparental care, behaviours which have the potential to reduce rates of multiple paternity [21]. Nevertheless, the available evidence does not support the assumption that external fertilization is necessarily associated with higher sperm competition levels than internal fertilizers.

At a broader taxonomic level, rates of multiple paternity in internally fertilizing fishes are equivalent to rates reported in reptiles, but higher than rates found in both birds and mammals [21]. The limited available data on female sperm storage duration across animals suggests that female fishes and reptiles typically store sperm for longer durations than birds and mammals [23]. As longer female sperm storage can increase sperm competition risk [24], the differences in rates of multiple paternity among these internally fertilizing taxa may reflect underlying variation in the duration of female sperm storage. Determining if female sperm storage duration covaries with rates of multiple paternity in fishes would be an interesting avenue for future investigation.

## 3. Sperm competition theory and fertilization mode in fishes

Sperm competition theory makes several testable predictions about how the selection is expected to act on sperm number, allocation, size and (albeit indirectly) sperm swimming speed, and longevity [13–17]. I briefly summarize these predictions here while highlighting how they could be affected by fertilization mode (table 1). I focus on predictions from raffle models, the most common class of models, which treat success during sperm competition as analogous to either a ‘fair’ (i.e. fertilization success is determined by the proportional representation of sperm from rival males) or ‘loaded’ (i.e. fertilization success is biased to favour of one of the competitors) raffle [17].

Sperm competition dynamics are influenced by four major factors [17], including (i) space constraints associated with competitive fertilizations, (ii) the degree of mixing between competing ejaculates, (iii) manipulation of ejaculates by either sex and (iv) inherent differences in fertilization probabilities among competing males. Each of these factors could be influenced by fertilization mode, albeit to varying degrees. External fertilizers are less likely to be influenced by space constraints than most (particularly small-bodied) internal fertilizers, assuming female sperm storage is limited in internal fertilizers [13,17]. Sperm mixing among competing ejaculates may also be more common in external fertilizers, as the external release and dilution of ejaculates likely prevents stratification or clumping effects among competing males. Arguably, ejaculate manipulation is more easily realized among internal fertilizers, where male genitalia can attempt to remove (or otherwise influence) rival sperm from the female's reproductive tract [21] and females have greater scope to influence sperm mixing, retention and performance (e.g. [25]) than in external fertilizers. Finally, intrinsic differences between internal and external fertilizers in the relative timing of gamete release prior to insemination may influence how mating order effects influence adaptive sperm allocation [21]. Thus, there is clearly scope for differences in sperm competition mechanisms to manifest between fertilization modes, although this has attracted scant empirical attention [10].

Predictions about how sperm competition influences sperm allocation and quality are sensitive to a range of assumptions, reflecting the biological complexity of reproduction observed in animals. These predictions may also be influenced by biological differences between fertilization modes (table 1). Generally, sperm allocation is expected to vary depending on the *risk* (the probability of competing with another ejaculate) and *intensity* (the number of ejaculates competing to fertilize a given set of eggs) of sperm competition ([17], table 1). Yet specific predictions for sperm allocation are sensitive to a wide range of moderators, including the amount of information a male possesses (e.g. about his phenotype, mating role, number of competitors [17]). Fertilization mode could influence how much information is available to males during mating: for example, external fertilizers may be better able to assess the number of competitors during spawning events, particularly compared with sequentially mating internal fertilizers, where female sperm storage dissociates mating from fertilization (table 1). Predictions for how sperm competition influences sperm quality are similarly complex and have the potential to vary with fertilization mode (table 1). Existing models focus on sperm size and do not deal with sperm swimming speed/longevity/viability directly. Instead, models make assumptions about the relationship between sperm size and correlated metrics of performance (e.g. swimming speed, longevity [14,15]). Therefore, assessing whether and how fertilization mode influences responses in sperm quality to sperm competition requires a better understanding of the functional link between sperm size and performance in external and internally fertilizing fishes (table 1).

## 4. Sperm competition in externally fertilizing fishes

The vast majority of the greater than 33 000 species of bony fishes reproduce using external fertilization, a broad term that describes the release of sperm and eggs into the external environment and ranges from broadcast spawning to the direct placement of sperm onto eggs. Below, I discuss how sperm competition influences sperm number, allocation and quality at the inter- and intra-specific level.

### (a) Sperm number in externally fertilizing fishes

Across species, there is robust support for the prediction that sperm number increases with sperm competition level in externally fertilizing fishes. Increases in relative testes size in response to increasing sperm competition levels have been reported across a range of fishes when using behavioural classification schemes based on mating systems (e.g. monogamous versus polyandrous) or mating behaviours (e.g. pair versus group spawning), with rare exceptions (reviewed by [6]). In a recent study that primarily assessed externally fertilizing fishes, Rowley *et al*. [22] found a clear link between relative testes size and estimates of sperm competition risk (rates of multiple paternity) and intensity (mean number of sires). This pattern was robust—positive correlations between testes size and sperm competition risk/intensity were evident when analyses were confined to cichlids and when male-pregnancy species were excluded from the analysis [22].

Within species, there is ample evidence that sperm number in a key determinant of male fertilization success and increases with sperm competition level in externally fertilizing fishes. Numerous studies demonstrate that male reproductive success increases with the number of sperm released/present during fertilizations (e.g. in several coral reef fishes [26], walleye, *Sander vitreus* [27], Atlantic cod, *Gadus morhua* [28]). Some of the best evidence that sperm competition selects for increased sperm number comes from comparisons of males with alternative reproductive tactics, where sneakers invest more in testicular tissue than guarding/parental males due to their elevated sperm competition risk (reviewed by [6,29]).

Early insights into sperm allocation came from intra-specific studies in coral reef fishes, where males released more sperm when mating with higher-quality (i.e. larger, more fecund) females and when matings involved sperm competition risk [26]. This work set the stage for a more detailed investigation of strategic sperm allocation in externally fertilizing fishes, which found that males release more sperm when a rival male is present, supporting predictions from sperm competition risk models (reviewed by [6]). Support for predictions from sperm competition intensity models comes from two externally fertilizing goby species, where sneaker males decrease sperm allocation when the intensity of sperm competition increases [30]. However, support for predictions of sperm allocation from the risk and intensity model are not universal in externally fertilizing fishes [6] and may depend on whether males mate in a favoured (i.e. guarding or dominant males) or a disfavoured (i.e. sneakers or subordinate males) role (e.g. [31,32]).

### (b) Sperm quality in externally fertilizing fishes

Externally fertilizing fish have proved a useful model for examining the link between sperm morphology and swimming performance both across and within species. Longer sperm swim faster across African cichlids and evolutionary modelling demonstrated that changes in female mating behaviour preceded evolutionary changes in sperm length and speed in these fishes [33]. This result offers clear support for the functional link between sperm length and speed and the importance of sperm competition in driving evolutionary changes in correlated ejaculate traits. Liao *et al*. [12] recently confirmed a positive relationship between sperm length and velocity across a broader range of fishes. The link between sperm size and swimming speed are less obvious (or not apparent at all) when examined intra-specifically [33,34]. One possible explanation for the disconnect between sperm size and speed is that most studies have not adequately accounted for the often-extensive intra-male variation present in ejaculate traits. When linking sperm length and swimming speed at the individual cell level, Simpson *et al*. [20] found that sperm with longer flagella and smaller head swam faster in rainbowfish (*Melanotaenia australis*).

How sperm competition influences sperm morphology and performance among species remains a point of contention in fishes. In an early and influential paper, Stockley *et al.* [11] reported a negative relationship between sperm length and sperm competition level, a finding that was not predicted by existing models. However, subsequent analyses that expanded Stockley *et al*.'s [11] dataset and excluded two species from the analysis (one with biflagellate spermatozoa and one extreme outlier) revealed a positive relationship between sperm length and sperm competition level across fishes [6]. Similarly, in African cichlids, both sperm length and swimming speed increase in response to elevated sperm competition levels [33,35]. While a consensus appeared to be emerging in fishes, three recent studies present an alternative view. Sperm velocity is not related with sperm competition level in mouth-brooding cichlids, where fertilization typically occurs within the confines of the female buccal cavity [36]. Moreover, Liao *et al*. [12] did not find a relationship between sperm length and sperm competition across fishes, although the authors note the species in their dataset likely face the opposing selective forces of sperm limitation and sperm competition. A recent study of West African riverine cichlids reported a negative relationship between sperm flagellum and head (but not midpiece) length and sperm competition risk [37]. Increased water velocity faced by riverine species was hypothesized to impact this relationship [37]. Indeed, fish spawning in more turbulent water release greater numbers of slower-swimming sperm that remain active for shorter durations, although spawning environment did not influence sperm morphology [12]. Sperm longevity can also be influenced by behavioural dynamics associated with fertilizations, as in the case of mouthbrooding cichlid species that spawn in bowers. In these cichlids, sperm release and fertilization are temporally separated and bower-building species produce longer-lived sperm than non-bower-building species [36].

There is a clear intra-specific link between sperm swimming speed and sperm competition in externally fertilizing fishes. Ample evidence from a range of species demonstrates that faster-swimming sperm sire a greater proportion of eggs in competitive *in vitro* experiments [2,38]. Work on salmonids also shows that sperm swimming speed responds rapidly to experimentally induced changes in male mating roles, with faster-swimming sperm produced by males in the disfavoured compared to favoured roles (e.g. [39,40]). The rapid changes in sperm swimming speed in salmonids are mediated by the non-sperm component of the ejaculate, the seminal fluid [40], similar to the seminal fluid-dependent sperm velocities observed in the grass goby (*Zosterisessor ophiocephalus* [41]). Sperm swimming speed also increases in response to social conditions indicating sperm competition risk in zebrafish, *Danio rerio* [42]. Surprisingly, however, differences in sperm swimming speed between dominant and sneaker males exhibits a mix of patterns in species with alternative reproductive tactics (reviewed by [29]), where differences in male mating roles between guarding and sneaker males are often linked with male phenotypes, more stable and arguably more extreme.

How sperm competition influences sperm morphology is unclear at the intra-specific level. Sperm morphology is rarely related with the outcome of competitive male fertilization success (e.g. [38]) and does not appear to differ between dominant and sneaker males in species with alternative reproductive tactics (reviewed by [29]). If sperm morphology is linked with sperm velocity in externally fertilizing species (e.g. [20]), why is sperm morphology generally uninformative in predicting competitive fertilization success? A detailed experiment in sticklebacks, *Gasterosteus aculeatus*, hints at an answer: sperm flagellum length predicts male fertilization success only under conditions when sperm and eggs interact for short periods of time, a result that emerges due to a trade-off between sperm speed and longevity in these fish [43]. When sperm and eggs are experimentally allowed to interact for longer time periods, smaller and longer-lived sperm fertilized more eggs [43]. Thus, the relative timing of sperm and egg release and the extent of gametic interactions are key factors to consider when examining the role of sperm size and speed in competitive matings of externally fertilizing fishes.

## 5. Sperm competition in internally fertilizing fishes

Internal fertilization is relatively rare in fishes, found only in basal cartilaginous fishes (class Chondrichthyes, figure 1) and in approximately 500 of the greater than 33 000 species of bony fishes (superclass Osteichthyes). Roughly 40% of internally fertilizing bony fishes are from the family Poeciliidae, a diverse group that are used commonly as a model for studying sperm competition [7]. The vast majority of research on sperm competition in internally fertilizing fishes has been conducted on poeciliids. By contrast, our understanding of how sperm competition influences the evolution of ejaculates remains in its infancy in Chondrichthyes. I discuss these two groups of internally fertilizing fishes separately below.

### (a) Sperm number and quality in sharks

Chondrichthyes (sharks, skates, rays and chimeras) are an ancient group of cartilaginous fishes that have lived and reproduced in the world's oceans since long before either trees or flowering plants evolved on land. Yet, research examining sperm competition in this ancient group has notably lagged behind other taxa [44]. The only available tests of predictions from sperm competition theory in sharks focus on sperm number (i.e. testes size) and sperm length and variance [22,45]. Specifically, male sharks invest more in relative testes mass when genetic estimates of greater sperm competition risk (percentage of broods sired by multiple males) and intensity (number of sires per brood) are higher [22]. Rowley *et al*.'s [45] analysis of sperm morphology from 25 shark species revealed that sperm flagellum (and total) length increased with sperm competition level, while within-male variance in flagellum length decreased. These findings offer clear evidence that sperm competition influenced the evolution of sperm number and morphology at the base of the vertebrate tree of life.

### (b) Sperm number and quality in internally fertilizing bony fishes

Selection on sperm number has been studied extensively in poeciliid fishes (family Poeciliidae [7]). Sperm number is the most important predictor of competitive fertilization success in the guppy, *Poecilia reticulata* [46]. However, recent work by Cattelan *et al*. [47] highlights the importance of taking a wider view when assessing responses to sperm competition. Working with guppies, Cattelan *et al*. [47] generated artificially selected lines of males with either high or low sperm production for three generations, leading to rapid divergence in sperm production between the lines. When they stopped artificially selecting on sperm production and reintroduced sexual selection the high and low sperm production lines converged to the sperm production of the original population within two generations. Responses in sperm number to selection, therefore, appear to be constrained in guppies, likely subject to trade-offs with other traits, which may limit evolutionary responses in sperm number [47]. Considering such limitation may help to explain why relative testes size and available sperm reserves does differ between sneaker and courter males in the swordtail *Xiphophorus nigrensis*, a species with alternative male reproductive tactics [48].

Across poeciliids, there is mixed support for how sperm competition risk and intensity influences sperm allocation. Males allocate more sperm when exposed to a single competitor (risk), but do not reduce sperm allocation when exposed to multiple competitors (intensity; reviewed by [6,7]). When assessing sperm swimming speed, poeciliids either show increases [49], no response [50] or decreases [51] when exposed to social conditions indicating sperm competition risk, and no response to sperm competition intensity [50]. Responses in sperm swimming speed to sperm competition risk can differ between male mating roles in species with alternative reproductive tactics (i.e. courters versus sneakers [49]), adding an additional layer of complexity to consider when assessing patterns of adaptive sperm allocation.

Artificial insemination has been used to investigate the importance of sperm swimming speed, viability and morphology on competitive fertilization success in poeciliids. Sperm swimming speed and viability routinely emerge as predictors of competitive fertilization success [46,52,53], although these effects can be mediated by the duration of sperm storage within the female's reproductive tract before their use [54–56] and by complex multivariate relationships with other reproductive traits [57]. Evidence of a link between sperm morphology and sperm competition in poeciliids comes from a comparison of sneaker and courter males in *X. nigrensis*, where sneaker males had larger midpieces (along with more viable sperm) than courter males [49]. Yet, there is currently no evidence that sperm morphology is related with competitive fertilization success in poeciliids [46,52–54], which may be due in part to a disconnect between sperm morphology and performance (e.g. [20]). Instead, complex multivariate relationships link sperm morphology (along with other male traits) to sperm swimming speed in guppies [58], suggesting that direct links between sperm morphology and competitive fertilization success may be challenging to detect. These complex relationships may be driven by genetic correlations between sperm quality and sexual ornaments [59] or among sperm quality traits (i.e. sperm morphology, swimming speed and viability [60]) that constrain responses to selection.

## 6. Conclusion and future directions

We can draw several general conclusions by comparing responses to sperm competition between fertilization modes using the available evidence summarized above.

1. Rates of multiple paternity, a proxy for sperm competition level, are higher in internally than externally fertilizing fishes. This unexpected finding may either arise from sampling bias or demonstrate the importance of prolonged female sperm storage in internal fertilers as a moderator of sperm competition levels in fishes.
2. In both fertilization modes, sperm number predicts competitive fertilization success and fishes respond to sperm competition by increasing investment in sperm number. This robust response in sperm number is evident at the intra- and interspecific level and mirrors the general pattern observed among animals [18,29].
3. Males typically respond to sperm competition risk by allocating more sperm to their ejaculates in both internal and external fertilizers. However, exceptions to this pattern are evident and can depend on male mating role, with males mating in disfavoured roles (e.g. sneaker males) showing greater plasticity in ejaculate allocation than males mating in favoured roles (e.g. guarding males). Males mating in different roles are likely privy to different amounts of information: favoured males may not know when they are being parasitized and therefore their sperm allocation strategy is tailored by mean sperm competition risk levels in the population, while disfavoured males may be aware of their mating role [21]. The predicted decrease in sperm allocation with increasing sperm competition intensity, however, has been reported only in externally fertilizing fishes. While further examples are required, this pattern suggests that externally fertilizing species may be better able to assess the number of potential sperm competitors than sequentially mating internal fertilizers. However, interpreting patterns of male allocation, particularly when testing sperm competition intensity models, requires careful consideration of total sperm reserves and ejaculated sperm number, as both of these ejaculate traits may respond to sperm competition levels. Overall, the observed pattern of sperm allocation in fishes matches closely with the general pattern observed across animals [19].
4. Sperm swimming speed routinely predicts competitive fertilization success when sperm number is held constant in internally and externally fertilizing fishes. However, it is important to note that artificial inseminations in internally fertilizing fishes typically involve the simultaneous insemination of sperm from two males, which may generate conditions that amplify the importance of sperm swimming speed in competitive fertilizations. Indeed, sperm velocity does not predict competitive fertilization success in guppies when artificial inseminations are temporally separated by 24 h [56].
5. Between fertilization modes, differences in how ejaculates respond to sperm competition are most apparent in sperm quality. Increases in sperm swimming speed in response to sperm competition risk are more apparent and consistent in external fertilizers than internal fertilizers.
6. The relationship between sperm morphology and performance remains unclear in fishes, and has yet to be assessed in large taxonomic groups (e.g. sharks and rays). Future empirical work should examine linear and nonlinear relationships between sperm size and swimming speed (in addition to other traits) in both externally and internally fertilizing species, which will aid in determining how/whether fertilization mode influences the relationship between sperm morphology and performance.
7. Across species, sperm size shows the mixed evolutionary response to sperm competition, particularly in external fertilizers. Fishes, therefore, continue to stand out from the general pattern of increasing sperm length in response to sperm competition observed in other taxa [53]. There are clear gaps in the literature, including the lack of comparative studies examining ejaculates in poeciliids (or other internally fertilizing bony fishes) and consideration of whether and how sperm competition influences within- and between-male variation in sperm morphology in externally fertilizing fishes. Addressing these gaps, and considering fertilization environments in greater detail in future studies, will help to clarify how selection differs between fertilization modes.
8. The importance of seminal fluid as a mediator of ejaculatory responses to variation in sperm competition is beginning to come into focus in fishes. Whether the role of seminal fluid differs based on fertilization mode remains an open question. Alongside increased focus on seminal fluids, investigating the evolutionary origins and significance of accessory glands, which produce substances that can influence sperm performance and are found in both externally and internally fertilizing fishes, in the context of sperm competition and fertilization mode would be illuminating.

Clarifying how fertilization mode influences the scope and potential for sperm competition to operate in fishes will require further research effort that probes at the different responses in ejaculates highlighted in this review. There is also the potential to take a broader view of post-copulatory sexual selection by contrasting how fertilization mode influences cryptic female choice in fishes. Interactions between ovarian fluid and sperm have the potential to alter selection on ejaculates and impact patterns of sperm evolution in both external (e.g. [61]) and internal (e.g. [25]) fertilizers. Finally, our understanding of how ejaculates respond to sperm competition is undoubtedly influenced by the species used to address this question. Expanding our gaze to the murkier waters of the fish tree of life will help advance our understanding of sperm competition in fishes and the importance of fertilization mode in shaping evolutionary responses in ejaculates across animals.

## Supplementary Material

Supplementary methods and table with multiple paternity data from 101 fishes.

## Data accessibility

All data are provided in the electronic supplementary material.
