# Supplementary material for: Sperm competition and fertilization mode in fishes
Source: Philos Trans R Soc Lond B Biol Sci. 2020 Oct 19;375(1813):20200074. doi: 10.1098/rstb.2020.0074 (PMC7661453; doi:10.1098/rstb.2020.0074)
Supplement: Supplementary methods and table with multiple paternity data from 101 fishes. [file rstb20200074supp1.docx]

**Supplementary Information**

**Sperm competition and fertilization mode in fishes**

John L. Fitzpatrick

Department of Zoology/Ethology, Stockholm University, Svante Arrhenius väg 18B, Stockholm 10691, Sweden

ORCID: 0000-0002-2834-4409

Correspondence: [john.fitzpatrick@zoologi.su.se](mailto:john.fitzpatrick@zoologi.su.se)

Running title: Sperm competition in fish

**Supplementary Methods**

To examine the effect of fertilization mode on rates of multiple paternity, I collected multiple paternity data (i.e. the proportion of nests/broods/litters where >1 males sire progeny) from the literature spanning a quarter of a century (Table S1). Species were only included if >1 nest/brood/litter were assayed using microsatellite loci to determine parentage. The resulting dataset included 101 species: 44 external fertilizers, 49 internal fertilizers, and 8 species with male pregnancy (Table S1). When estimates of multiple paternity were available from more than one study or population, I calculated sample size-weighted mean values for use in subsequent analyses. To place this data in a phylogenetic framework, I downloaded a distribution of 10,000 trees from cartilaginous fishes (class Chondrichthyes) including 1192 species from <http://vertlife.org/sharktree/> and a consensus phylogeny of a 11,638 species of bony fishes (superclass Osteichthyes) from <https://fishtreeoflife.org>. I generated a consensus tree from the distribution of trees from Chondrichthyes using TreeAnnotator (v.1.10.4) in BEAST [1], with the burnin set to 1000, posterior probability limit set to 0.5 and using posterior medians for node heights. Consensus phylogenies were then imported into RStudio (v1.1.453) where all subsequent manipulation and analysis took place. I grafted the Chondrichthyes and Osteichthyes consensus trees together using divergence estimates from the Timetree of life (<http://www.timetree.org>), setting the point of nodal divergence between these groups at 473 million years ago. This generated a fish-wide phylogeny including both cartilaginous and bony fishes. However, this process generated some scaling issues, which were resolved by collapsing negative branches and ensuring the final phylogeny was ultrametric.

I then matched the dataset with species represented in the fish-wide phylogeny. Of the 101 species in the dataset, 93 were present in the fish-wide phylogeny. Three species that were not present in the fish-wide phylogeny (*Mchenga eucinostomus*, *Pseudotropheus zebra*, *Symphodus ocellatus*) were included by assigning them the phylogenetic position of species from the same genus that were present in the fish-wide phylogeny. Two *Xiphophorus* species (*X. birchmanni* and *X. multilineatus*) were placed in the fish-wide phylogeny by assigning them the phylogenetic position of closely related species (*X. malinche* and *X. continens*, respectively, with phylogenetic positions based on [2]) to maintain the evolutionary position of these fishes with *Xiphophorus* species already present in the fish-wide phylogeny. This increased the total number of species included in the phylogeny to 98. Note that three externally fertilizing species (*Protomelas c.f. spilopterus*, *Pseudotropheus ‘mazinzi blue’*, *Pseudotropheus c.f gracilor*) were not placed in the final fish-wide phylogeny.

Fertilization mode was reconstructed on the phylogeny using the make.simmap function in phytools [3], in a model specifying different evolutionary rates of gains and losses in fertilization mode (i.e. all rates different, or ARD). I then performed a phylogenetically controlled generalized least-squared (PGLS) regression to examine if rates of multiple paternity (transformed using log10(x+1) values) differed between fertilizations modes while accounting for the evolutionary relationships among species [4]. PGLS models estimate a phylogenetic scaling parameter, λ, that assesses the degree of phylogenetic dependent in the data and ranges from zero (phylogenetic independence) to one (phylogenetic dependence) [4]. Species with male pregnancy were excluded from analyses contrasting fertilization mode, as the reproductive biology of these species is unusual compared to other fishes and they exhibit no variance in rates of multiple paternity among the species included in the analysis.

**Table S1. Fertilization mode and rates of multiple paternity in (a) bony fishes (superclass Osteichthyes) and (b) cartilaginous fishes (class Chondrichthyes).** Data on fertilization mode and multiple paternity was collected for 101 species. Fertilization mode is categorized as external and internal fertilization. Sex-role reversed seahorses and pipefish species are listed as male pregnancy species, and these fishes were excluded from comparisons of multiple paternity between fertilization modes. When estimates of multiple paternity were available from multiple studies or populations, sample size-weighted mean values were calculated and are presented here.

|  | **Species** | **Fertilization mode** | **Multiple Paternity (%)** | **References** |
| --- | --- | --- | --- | --- |
| ***(a) Bony fishes (superclass Osteichthyes)*** | | |  |  |
|  | *Abudefduf sordidus* | External | 14.7 | 5 |
|  | *Aphredoderus sayanus* | External | 100 | 6 |
|  | *Clinus cottoides* | Internal | 100 | 7-8 |
|  | *Clinus supercilosus* | Internal | 83 | 8 |
|  | *Cottus bairdi* | External | 0 | 9 |
|  | *Ctenochromis horei* | External | 57 | 10 |
|  | *Cymatogaster aggregata* | Internal | 91.9 | 11-12 |
|  | *Cyprichromis coloratus* | External | 33.3 | 13 |
|  | *Cyprichromis leptosoma* | External | 81.8 | 13 |
|  | *Embiotoca jacksoni* | Internal | 100 | 14 |
|  | *Embiotoca lateralis* | Internal | 100 | 14 |
|  | *Eretmodus cyanostictus* | External | 0 | 15 |
|  | *Etheostoma olmstedi* | External | 6 | 16 |
|  | *Etheostoma virgatum* | External | 0 | 17 |
|  | *Gambusia affinis* | Internal | 73.4 | 18 |
|  | *Gambusia holbrooki* | Internal | 61.9 | 18-20 |
|  | *Gasterosteus aculeatus* | External | 20.2 | 21-22 |
|  | *Gobiusculus flavescens* | External | 5 | 23 |
|  | *Heterandria formosa* | Internal | 46.1 | 24 |
|  | *Hexagrammos otackii* | External | 100 | 25 |
|  | *Hippocampus abdominalis* | Male Pregnancy | 0 | 26 |
|  | *Hippocampus subelongatus* | Male Pregnancy | 0 | 27-28 |
|  | *Hyperprosopon anale* | Internal | 100 | 11 |
|  | *Hysterocarpus traski* | Internal | 92 | 29 |
|  | *Ictalurus punctatus* | External | 0 | 30 |
|  | *Julidochromis ornatus* | External | 23 | 31 |
|  | *Lepomis auritus* | External | 12 | 32 |
|  | *Lepomis gibbosus* | External | 53.8 | 33-34 |
|  | *Lepomis macrochirus* | External | 94.9 | 33,35 |
|  | *Lepomis marginatus* | External | 4 | 36 |
|  | *Lepomis punctatus* | External | 40 | 37 |
|  | *Leuresthes tenuis* | External | 88 | 38 |
|  | *Mchenga cyclicos* | External | 100 | 39 |
|  | *Micropterus salmoides* | External | 4 | 40 |
|  | *Muraenoclinus dorsalis* | Internal | 78.5 | 8, 41 |
|  | *Neolamprologus caudopunctatus* | External | 0 | 42 |
|  | *Neolamprologus modestus* | External | 66.7 | 43 |
|  | *Neolamprologus pulcher* | External | 50.8 | 44-47 |
|  | *Nerophis ophidion* | Male Pregnancy | 0 | 48 |
|  | *Ophiodon elongatus* | External | 62 | 49 |
|  | *Osteoglossum bicirrhosum* | External | 8.3 | 50 |
|  | *Petrochromis fasciolatus* | External | 50 | 51 |
|  | *Pimephales promelas* | External | 17 | 52 |
|  | *Poecilia latipinna* | Internal | 70 | 53 |
|  | *Poecilia reticulata* | Internal | 63.8 | 55-57 |
|  | *Pomatoschistus minutus* | External | 45 | 58-59 |
|  | *Porichthys notatus* | External | 100 | 60 |
|  | *Protomelas c.f. spilopterus* | External | 50 | 61 |
|  | *Pseudotropheus ‘mazinzi blue’* | External | 100 | 39 |
|  | *Pseudotropheus c.f gracilor* | External | 67 | 39 |
|  | *Pseudotropheus zebra* | External | 86 | 62 |
|  | *Salmo salar* | External | 36.3 | 63-66 |
|  | *Salvelinus fontinalis* | External | 20 | 67 |
|  | *Scartella cristata* | External | 83 | 68 |
|  | *Sebastes alutus* | Internal | 71.2 | 69 |
|  | *Sebastes atrovirens* | Internal | 100 | 70 |
|  | *Sebastes inermis* | Internal | 20 | 71 |
|  | *Sebastes maliger* | Internal | 32 | 72 |
|  | *Sebastes schlegelii* | Internal | 90.9 | 73 |
|  | *Sphaeramia nematoptera* | External | 7.6 | 74 |
|  | *Spinachia spinachia* | External | 18 | 75 |
|  | *Symphodus ocellatus* | External | 100 | 76 |
|  | *Syngnathus auliscus* | Male Pregnancy | 0 | 77 |
|  | *Syngnathus floridae* | Male Pregnancy | 0 | 78-80 |
|  | *Syngnathus leptorhynchus* | Male Pregnancy | 0 | 77, 81 |
|  | *Syngnathus scovelli* | Male Pregnancy | 0 | 82-83 |
|  | *Syngnathus typhle* | Male Pregnancy | 0 | 84-85 |
|  | *Telmatochromis temporalis* | External | 29 | 86 |
|  | *Tropheus moorii* | External | 0 | 51 |
|  | *Variabilichromis moorii* | External | 82.8 | 87-88 |
|  | *Xiphophorus birchmanni* | Internal | 83.7 | 89 |
|  | *Xiphophorus helleri* | Internal | 62.8 | 90-91 |
|  | *Xiphophorus multilineatus* | Internal | 28 | 92 |
|  | *Xiphophorus nigrensis* | Internal | 61.3 | 93 |
|  |  |  |  |  |
| ***(b) Cartilaginous fishes (class Chondrichthyes)*** | | |  |  |
|  | *Carcharhinus amblyrhynchos* | Internal | 66 | 94 |
|  | *Carcharhinus leucas* | Internal | 55 | 95-96 |
|  | *Carcharhinus limbatus* | Internal | 50 | 97 |
|  | *Carcharhinus obscurus* | Internal | 35 | 98 |
|  | *Carcharhinus plumbeus* | Internal | 52.5 | 99-100 |
|  | *Carcharias taurus* | Internal | 40 | 101 |
|  | *Galeocerdo cuvier* | Internal | 0 | 102 |
|  | *Galeorhinus galeus* | Internal | 40 | 103 |
|  | *Isurus oxyrinchus* | Internal | 100 | 104 |
|  | *Mustelus antarcticus* | Internal | 31 | 105 |
|  | *Mustelus asterias* | Internal | 83 | 106 |
|  | *Mustelus henlei* | Internal | 93 | 107-108 |
|  | *Mustelus lenticulatus* | Internal | 42 | 105 |
|  | *Mustelus mustelus* | Internal | 47 | 109 |
|  | *Mustelus punctulatus* | Internal | 54 | 109 |
|  | *Negaprion acutidens* | Internal | 78 | 110 |
|  | *Negaprion brevirostris* | Internal | 85.6 | 111-113 |
|  | *Prionace glauca* | Internal | 80 | 114 |
|  | *Raja clavate* | Internal | 100 | 115 |
|  | *Rhizoprionodon terraenovae* | Internal | 40 | 116 |
|  | *Scyliorhinus canicula* | Internal | 92 | 117 |
|  | *Sphyrna lewini* | Internal | 61 | 94,98 |
|  | *Sphyrna tiburo* | Internal | 18.8 | 118 |
|  | *Squalus acanthias* | Internal | 20.3 | 119-120 |
|  | *Squalus mitsukurii* | Internal | 11.1 | 121 |
|  | *Triakis semifasciata* | Internal | 36.4 | 122 |
|  | *Urobatis halleri* | Internal | 90 | 123 |

**References**

1. Suchard MA, Lemey P, Baele G, Ayres DL, Drummond AJ, Rambaut A. 2018 Bayesian phylogenetic and phylodynamic data integration using BEAST 1.10. *Virus Evol*. 4, vey016. (doi:10.1093/ve/vey016)
2. Culumber ZW, Tobler M. 2016 Ecological divergence and conservatism: spatiotemporal patterns of niche evolution in a genus of livebearing fishes (Poeciliidae: *Xiphophorus*). *BMC Evol. Biol*. 16, 44. (doi:10.1186/s12862-016-0593-4)
3. Revell, L. J. 2012. Phytools: an R package for phylogenetic comparative biology (and other things). *Methods Ecol. Evol.* 3, 217-223. (doi:10.1111/j.2041-210X.2011.00169.x)
4. Freckleton RP, Harvey PH, Pagel M. 2002 Phylogenetic analysis and comparative data: a test and review of evidence. *Am. Nat*. 160, 712‐726. (doi:10.1086/343873)
5. Kerr Lobel L, Drown DM, Barber PH, Lobel PS. 2019 A Genetic Assessment of Parentage in the Blackspot Sergeant Damselfish, *Abudefduf sordidus* (Pisces: Pomacentridae). *Fishes*. 4, 53. ([doi.org/10.3390/fishes4040053](https://doi.org/10.3390/fishes4040053))
6. Fletcher DE, Dakin EE, Porter BA, Avise JC. 2004 Spawning Behavior and Genetic Parentage in the Pirate Perch (*Aphredoderus sayanus*), a fish with an enigmatic reproductive morphology. *Copeia* 1, 1-10. (doi:10.1643/CE-03-160R)
7. Scheepers, M.J., Gouws, G. & Gon, O. 2018 Evidence of multiple paternity in the bluntnose klipfish, *Clinus cottoides* (Blennioidei: Clinidae: Clinini). *Environ. Biol. Fish.* 101, 1669–1675. (doi.org/10.1007/s10641-018-0815-2)
8. Scheepers MJ, Gouws G. 2019 Mating system, reproductive success, and sexual selection in bluntnose klipfishes (*Clinus cottoides*). *J. Hered*. **110**, 351‐360. (doi:10.1093/jhered/esz008)
9. Fiumera AC, Porter BA, Grossman GD, Avise JC. 2002 Intensive genetic assessment of the mating system and reproductive success in a semi-closed population of the mottled sculpin, *Cottus bairdi*. *Mol. Ecol*. **11**, 2367‐2377. (doi:10.1046/j.1365-294x.2002.01585.x)
10. Sefc KM, Hermann CM, Koblmüller S. 2009 Mating system variability in a mouthbrooding cichlid fish from a tropical lake. *Mol. Ecol*. **18**, 3508‐3517. (doi:10.1111/j.1365-294X.2009.04295.x)
11. LaBrecque JR, Alva-Campbell YR, Archambeault S, Crow KD. 2014 Multiple paternity is a shared reproductive strategy in the live-bearing surfperches (Embiotocidae) that may be associated with female fitness. *Ecol. Evol*. **4**, 2316‐2329. (doi:10.1002/ece3.1071)
12. Liu JX, Avise JC. 2011 High degree of multiple paternity in the viviparous Shiner Perch, *Cymatogaster aggregata*, a fish with long-term female sperm storage. *Mar Biol*. **158**, 893‐901. (doi:10.1007/s00227-010-1616-0)
13. Anderson C, Werdenig A, Koblmüller S, Sefc KM. 2015 Same school, different conduct: rates of multiple paternity vary within a mixed-species breeding school of semi-pelagic cichlid fish (*Cyprichromis spp.*). *Ecol. Evol*. **6**, 37‐45. (doi:10.1002/ece3.1856)
14. Reisser CM, Beldade R, Bernardi G. 2009 Multiple paternity and competition in sympatric congeneric reef fishes, *Embiotoca jacksoni* and *E. lateralis*. *Mol. Ecol*. **18**, 1504‐1510. (doi:10.1111/j.1365-294X.2009.04123.x)
15. Taylor MI, Morley JI, Rico C, Balshine S. 2003 Evidence for genetic monogamy and female-biased dispersal in the biparental mouthbrooding cichlid *Eretmodus cyanostictus* from Lake Tanganyika. *Mol. Ecol.* **12**, 3173–3177. (doi:10.1046/j.1365-294x.2003.01964.x)
16. DeWoody JA, Fletcher DE, Wilkins SD, Avise JC. 2000. Parentage and nest guarding in the tessellated darter (*Etheostoma olmstedi*) assayed by microsatellite markers (Perciformes: Percidae). *Copeia* **2000**, 740–747. (doi:10.1643/0045-8511(2000)000[0740:PANGIT]2.0.CO;2)
17. Porter BA, Fiumera AC, Avise JC. 2002. Egg mimicry and allopaternal care: two mate attracting tactics by which nesting striped darter (*Etheostoma virgatum*) males enhance reproductive success. *Behav. Ecol. Sociobiol* **51**, 350–359. (doi.org/10.1007/s00265-002-0456-4)
18. Gao J, Santi F, Zhou L, Wang X, Riesch R, Plath M. 2019 Geographical and temporal variation of multiple paternity in invasive mosquitofish (*Gambusia holbrooki*, *Gambusia affinis*). *Mol. Ecol*. **28**, 5315‐5329. (doi:10.1111/mec.15294)
19. Zane, L., Nelson, W.S., Jones, A.G. & Avise, J.C. 1999. Microsatellite assessment of multiple paternity in natural populations of a live-bearing fish, *Gambusia holbrooki*. *J. Evol. Biol.* 12, 61–69. (doi:10.1046/j.1420-9101.1999.00006.x)
20. Zeng, Y., Díez-del-Molino, D., Vidal, O. *et al.* 2017 Multiple paternity and reproduction opportunities for invasive mosquitofish. *Hydrobiol.* 795, 139–151. (<https://doi.org/10.1007/s10750-017-3125-3>)
21. Largiadèr CR, Fries V, Bakker TC. 2001 Genetic analysis of sneaking and egg-thievery in a natural population of the three-spined stickleback (*Gasterosteus aculeatus* L.). *Hered.* 86, 459‐468. (doi:10.1046/j.1365-2540.2001.00850.x)
22. Blais J, Rico C, Bernatchez L. 2004 Nonlinear effects of female mate choice in wild threespine sticklebacks. *Evol.* **58**, 2498‐2510. (doi:10.1111/j.0014-3820.2004.tb00879.x)
23. Mobley KB, Amundsen T, Forsgren E, Svensson PA, Jones AG. 2009 Multiple mating and a low incidence of cuckoldry for nest-holding males in the two-spotted goby, *Gobiusculus flavescens*. *BMC Evol. Biol*. **9**, 6. (doi:10.1186/1471-2148-9-6)
24. Soucy S, Travis J. 2003 Multiple paternity and population genetic structure in natural populations of the poeciliid fish, *Heterandria formosa*. *J. Evol. Biol*. **16**, 1328‐1336. (doi:10.1046/j.1420-9101.2003.00608.x)
25. Munehara H, Takenaka O. 2000 Microsatellite markers and multiple paternity in a paternal care fish, *Hexagrammos otakii*. *J. Ethol.* **18,**101–104 (<https://doi.org/10.1007/s101640070007>)
26. Wilson AB, Martin-Smith KM. 2007 Genetic monogamy despite social promiscuity in the pot-bellied seahorse (*Hippocampus abdominalis*). *Mol. Ecol*. **16**, 2345‐2352. (doi:10.1111/j.1365-294X.2007.03243.x)
27. Jones AG, Kvarnemo C, Moore GI, Simmons LW, Avise JC. 1998 Microsatellite evidence for monogamy and sex-biased recombination in the Western Australian seahorse *Hippocampus angustus*. *Mol. Ecol*. **7**, 1497‐1505. (doi:10.1046/j.1365-294x.1998.00481.x)
28. Kvarnemo C, Moore GI, Jones AG, Nelson WS, Avise JC. 2000. Monogamous pair bonds and mate switching in the Western Australian seahorse *Hippocampus subelongatus*. *J. Evol. Biol.* **13,** 882–888. (doi:10.1046/j.1420-9101.2000.00228.x)
29. Liu JX, Tatarenkov A, O'Rear TA, Moyle PB, Avise JC. 2013 Molecular evidence for multiple paternity in a population of the viviparous Tule Perch *Hysterocarpus traski*. *J. Hered*. **104**, 217‐222. (doi:10.1093/jhered/ess105)
30. Tatarenkov A, Barreto F, Winkelman DL, Avise JC. 2006. Genetic monogamy in the channel catfish, *Ictalurus punctatus*, a species with uniparental nest guarding. *Copeia* **2006**, 735–741. (doi:10.1643/0045-8511(2006)6[735:GMITCC]2.0.CO;2)
31. Awata S, Munehara H, Kohda M. 2005 Social system and reproduction of helpers in a cooperatively breeding cichlid fish (*Julidochromis ornatus*) in Lake Tanganyika: field observations and parentage analyses. *Behav. Ecol. Sociobiol.* **58,**506–516. (doi:10.1007/s00265-005-0934-6)
32. DeWoody JA, Fletcher DE, Wilkins SD, Nelson WS, Avise JC. 1998. Molecular genetic dissection of spawning, parentage, and reproductive tactics in a population of redbreast sunfish, *Lepomis auritus*. *Evolution* **52**, 1802–1810. (doi:10.1111/j.1558-5646.1998.tb02257.x)
33. Neff BD, Clare EL. 2008 Temporal variation in cuckoldry and paternity in two sunfish species (*Lepomis spp.*) with alternative reproductive tactics. *Can. J. Zool.* **86**, 92–98. (doi:10.1139/Z07-121)
34. Rios-Cardenas O, Webster MS. 2005. Paternity and paternal effort in the pumpkinseed sunfish. *Behavioral Ecology* **16**, 914–921. (doi:10.1093/beheco/ari076)
35. Neff BD. 2001 Genetic paternity analysis and breeding success in bluegill sunfish (*Lepomis macrochirus*). *J. Hered*. **92**, 111‐119. (doi:10.1093/jhered/92.2.111)
36. Mackiewicz M, Fletcher DE, Wilkins D, DeWoody A, Avise JC. 2002. A genetic assessment of parentage in a natural population of dollar sunfish (*Lepomis marginatus*) based on microsatellite markers. *Mol. Ecol.* **11**, 1877–1883. (doi:10.1046/j.1365-294X.2002.01577.x)
37. Dewoody JA, Fletcher DE, MacKiewicz M, Wilkins SD, Avise JC. 2000 The genetic mating system of spotted sunfish (*Lepomis punctatus*): mate numbers and the influence of male reproductive parasites. *Mol. Ecol*. **9**, 2119‐2128. (doi:10.1046/j.1365-294x.2000.01123.x)
38. Byrne RJ, Avise JC. 2009 Multiple paternity and extra- group fertilizations in a natural population of California grunion (*Leuresthes tenuis*), a beach-spawning marine fish. *Mar. Biol.* 156: 1681–1690. (doi:10.1007/s00227-009-1203-4)
39. Kellogg KA, Markert JA, Stauffer JR Jr, Kocher TD. 1995 Microsatellite variation demonstrates multiple paternity in lekking fishes from Lake Malawi, Africa. *Proc. R. Soc. Lond. B*. **260**, 79–84. (doi:10.1098/rspb.1995.0062)
40. DeWoody JA, Fletcher DE, Wilkins SD, Nelson WS, Avise JC. 2000 Genetic monogamy and biparental care in an externally fertilizing fish, the largemouth bass (*Micropterus salmoides*). *Proc. R. Soc. Lond. B*. **267**, 2431‐2437. (doi:10.1098/rspb.2000.1302)
41. Schulze MJ, Henriques R, Feldheim KA, Bowie RCK, von der Heyden S. 2018 How many daddies: microsatellite genotyping reveals polyandry in a live-bearing clinid fish *Muraenoclinus dorsalis*. *J. Fish Biol*. **92**, 1435‐1445. (doi:10.1111/jfb.13598)
42. Schaedelin FC, van Dongen WFD, Wagner RH. 2015 Mate choice and genetic monogamy in a biparental, colonial fish. *Behav. Ecol.* **26**, 782–788. (doi:10.1093/beheco/arv011)
43. Hellmann JK, O'Connor CM, Ligocki IY, Farmer TM, Arnold TJ, Reddon AR, Garvy KA, Marsh‐Rollo SE, Balshine S and Hamilton, I.M. 2015 Alternative male morphs in *Neolamprologus modestus*. J. Zool. **296**, 116-123. (doi:10.1111/jzo.12222)
44. Stiver KA, Fitzpatrick JL, Desjardins JK, Balshine S. 2009 Mixed parentage in *Neolamprologus pulcher* groups. *J. Fish Biol*. **74**, 1129‐1135. (doi:10.1111/j.1095-8649.2009.02173.x)
45. Dierkes P, Taborsky M, Achmann R. 2008 Multiple paternity in the cooperatively breeding fish *Neolamprologus pulcher* . *Behav. Ecol. Sociobiol.* **62,**1581. (https://doi.org/10.1007/s00265-008-0587-3)
46. Bruintjes R, Bonfils D, Heg D, Taborsky M. 2011 Paternity of subordinates raises cooperative effort in cichlids. *PLoS One*. **6**, e25673. (doi:10.1371/journal.pone.0025673)
47. Hellmann JK, Sovic MG, Gibbs HL, et al. 2016 Within-group relatedness is correlated with colony-level social structure and reproductive sharing in a social fish. *Mol. Ecol*. **25**, 4001‐4013. (doi:10.1111/mec.13728)
48. McCoy EE, Jones AG, Avise JC. 2001 The genetic mating system and tests for cuckoldry in a pipefish species in which males fertilize eggs and brood offspring externally. *Mol. Ecol*. **10**, 1793‐1800. (doi:10.1046/j.0962-1083.2001.01320.x)
49. Withler RE, King JR, Marliave JB, Beaith B, Li S, Supernault KJ, Miller KM. 2004. Polygamous mating and high levels of genetic variation in lingcod, *Ophiodon elongatus*, of the Strait of Georgia, British Columbia. *Environ. Biol. Fish.* **69**, 345–357. (doi:10.1007/978-94-007-0983-6_28)
50. Verba JT, Neto JGR, Zuanon J, Farias I. 2014 Evidence of multiple paternity and cooperative parental care in the so called monogamous silver arowana *Osteoglossum bicirrhosum* (Osteoglossiformes: Osteoglossidae). Neotrop. Ichthy. **12**, 145-151. ([doi:10.1590/S1679-62252014000100015](https://dx.doi.org/10.1590/S1679-62252014000100015))
51. Egger B, Obermüller B, Phiri H, Sturmbauer C, Sefc KM. 2006 Monogamy in the maternally mouthbrooding Lake Tanganyika cichlid fish *Tropheus moorii*. *Proc. R. Soc. Lond. B*. **273**, 1797‐1802. (doi:10.1098/rspb.2006.3504)
52. Bessert ML, Brozek J, Ortí G. 2007 Impact of nest substrate limitations on patterns of illegitimacy in the fathead minnow, *Pimephales promelas* (Cypriniformes: Cyprinidae). *J. Hered*. **98**, 716‐722. (doi:10.1093/jhered/esm092)
53. Girndt A, Riesch R, Schröder C, Schlupp I, Plath M, Tiedemann R. 2012. Multiple paternity in different populations of the sailfin molly, *Poecilia latipinna*. *An. Biol.* **62**, 245-262. (doi: 10.1163/157075611X618192)
54. Hain TJA, Neff BD. 2007 Multiple paternity and kin recognition mechanisms in a guppy population. *Mol. Ecol.* **16**, 3938–3946. (doi:10.1111/j.1365-294X.2007.03443.x)
55. Elgee KE, Ramnarine IW, Pitcher TE. 2012 Multiple paternity, reproductive skew and correlates of male reproductive success in a wild population of the Trinidadian guppy. *Ecol. Freshw. Fish.* **21**, 109–118. (doi:10.1111/j.1600-0633.2011.00528.x)
56. Neff BD, Pitcher TE, Ramnarine IW. 2008 Inter-population variation in multiple paternity and reproductive skew in the guppy. *Mol. Ecol*. **17**, 2975‐2984. (doi:10.1111/j.1365-294X.2008.03816.x)
57. Kelly CD, Godin J-GJ. Wright JM. 1999 Geographic variation in multiple paternity within natural populations of the guppy (*Poecilia reticulata*). *Proc. R. Soc. Lond. B*. **266**, 2403–2408. (doi:10.1098/rspb.1999.0938)
58. Jones AG, Walker D, Kvarnemo C, Lindström K, Avise JC. 2001 How cuckoldry can decrease the opportunity for sexual selection: data and theory from a genetic parentage analysis of the sand goby, *Pomatoschistus minutus*. *Proc. Natl. Acad. Sci. U.S.A.* **98**, 9151–6. (doi:10.1073/pnas.171310198)
59. Jones AG, Walker D, Lindström K, Kvarnemo C, Avise JC. 2001 Surprising similarity of sneaking rates and genetic mating patterns in two populations of sand goby experiencing disparate sexual selection regimes. *Mol. Ecol*. **10**, 461‐469. (doi:10.1046/j.1365-294x.2001.01193.x)
60. Cogliati, K.M., Neff, B.D. & Balshine, S. 2013. High degree of paternity loss in a species with alternative reproductive tactics. *Behav. Ecol. Sociobiol.* **67**, 399–408. (https://doi.org/10.1007/s00265-012-1460-y)
61. Kellogg KA, Markert JA, Stauffer JR Jr, Kocher TD. 1998 Intraspecific brood mixing and reduced polyandry in a maternal mouth-brooding cichlid. *Behav. Ecol.* **9**, 309–312. (doi:10.1093/beheco/9.3.309)
62. Parker A, Kornfield I. 1996. Polygynandry in *Pseudotropheus zebra*, a cichlid fish from Lake Malawi. *Environ. Biol. Fish.* **47**, 345–352. (https://doi.org/10.1007/BF00005049)
63. Grimardias D, Merchermek N, Manicki A, Garnier J, Gaudin P, Jarry M, Beall E. 2010 Reproductive success of Atlantic salmon (*Salmo salar*) mature male parr in a small river, the Nivelle: influence of shelters. *Ecol. Freshw. Fish* **19**, 510–519. (doi:10.1111/j.1600-0633.2010.00421.x)
64. Weir LK, Breau C, Hutchings JA, Cunjak RA. 2010 Multiple paternity and variance in male fertilization success within Atlantic salmon *Salmo salar* redds in a naturally spawning population. *J. Fish Biol.* **77**, 479– 493. (doi:10.1111/j.1095-8649.2010.02690.x)
65. Taggart JB, McLaren IS, Hay DW, Webb JH, Youngson AF. 2001. Spawning success in Atlantic salmon (*Salmo salar* L.): a long-term DNA profiling-based study conducted in a natural stream. *Mol. Ecol.* **10**, 1047–1060. (doi:10.1046/j.1365-294x.2001.01254.x)
66. Martinez JL, Moran P, Perez J, De Gaudemar B, Beall E, Garcia‐Vazquez E. 2000 Multiple paternity increases effective size of southern Atlantic salmon populations. Mol. Ecol. **9**, 293-298. (doi:10.1046/j.1365-294x.2000.00857.x)
67. Blanchfield PJ, Ridgway MS, Wilson CC. 2003 Breeding success of male brook trout (*Salvelinus fontinalis*) in the wild. *Mol. Ecol*. **12**, 2417‐2428. (doi:10.1046/j.1365-294x.2003.01917.x)
68. Mackiewicz M, Porter BA, Dakin EE, Avise JC. 2005. Cuckoldry rates in the molly miller (*Scartella cristata*; Blenniidae), a hole nesting marine fish with alternative reproductive tactics. *Mar. Biol.* **148**, 213–221. (doi:10.1007/s00227-005-0010-9)
69. Van Doornik DM, Parker SJ, Millard SR, Berntson EA, Moran P. 2008. Multiple paternity is prevalent in Pacific ocean perch (*Sebastes alutus*) off the Oregon coast, and is correlated with female size and age. *Environ. Biol. Fish.* **83**, 269–275. (doi.org/10.1007/s10641-008-9331-0)
70. Sogard SM, Gilbert-Horvath E, Anderson EC, Fisher R, Berkeley SA, Garza JC. 2008 Multiple paternity in viviparous kelp rockfish, *Sebastes atrovirens*. *Environ. Biol. Fish.* **81**, 7–13. (doi.org/10.1007/s10641-006-9170-9)
71. Blanco Gonzalez E, Murakami T, Teshima Y, Yoshioka K, Jeong D-S, Umino T. 2009 Paternity testing of wild black rockfish *Sebastes inermis* (brownish type) from the Seto Inland Sea of Japan. *Ichthyol. Res.* **56**, 87–91. (doi.org/10.1007/s10228-008-0055-0)
72. Gray AK, Rodgveller CJ, Lunsford CR. 2015 Evidence of multiple paternity in quillback rockfish (*Sebastes maliger*). NOAA Technical Memorandum NMFS-AFSC-303 (doi:10.7289/V5PZ56T3)
73. Gao, T., Ding, K., Song, N. *et al.* 2018 Comparative analysis of multiple paternity in different populations of viviparous black rockfish, *Sebastes schlegelii*, a fish with long-term female sperm storage. *Mar. Biodiv.* **48**, 2017–2024. (https://doi.org/10.1007/s12526-017-0713-4)
74. Rueger T, Harrison HB, Gardiner NM, Berumen ML, Jones GP. 2019 Extra-pair mating in a socially monogamous and paternal mouth-brooding cardinalfish. *Mol. Ecol*. **28**, 2625‐2635. (doi:10.1111/mec.15103)
75. Jones AG, Östlund-Nilsson S, Avise JC. 1998 A microsatellite assessment of sneaked fertilizations and egg thievery in the fifteen spine stickleback. *Evol.* **52**, 848–858. (doi:10.1111/j.1558-5646.1998.tb03709.x)
76. Alonzo SH, Heckman KL. 2010 The unexpected but understandable dynamics of mating, paternity and paternal care in the ocellated wrasse. *Proc. R. Soc. Lond. B*. **277**, 115–122. (doi:10.1098/rspb.2009.1425)
77. Wilson AB. 2006. Interspecies mating in sympatric species of *Syngnathus* pipefish. *Mol. Ecol.* **15**, 809–824. (doi:10.1111/j.1365-294X.2006.02831.x)
78. Mobley KB, Jones AG. 2007 Geographical variation in the mating system of the dusky pipefish (*Syngnathus floridae*). *Mol. Ecol*. **16**, 2596‐2606. (doi:10.1111/j.1365-294X.2007.03337.x)
79. Mobley, K.B. & Jones, A.G. 2009. Environmental, demographic, and genetic mating system variation among five geographically distinct dusky pipefish (*Syngnathus floridae*) populations. *Mol. Ecol.* **18**: 1476–1490. (doi:10.1111/j.1365-294X.2009.04104.x)
80. Jones AG, Avise JC. 1997 Polygynandry in the dusky pipefish *Syngnathus floridae* revealed by microsatellite DNA markers. *Evol.* **51**, 1611–1622. (doi:10.1111/j.1558-5646.1997.tb01484.x)
81. Wilson AB. 2009 Fecundity selection predicts Bergmann’s rule in syngnathid fishes. *Mol. Ecol.* **18**, 1263–1272. (doi:10.1111/j.1365-294X.2009.04084.x)
82. Jones AG, Avise JC. 1997 Microsatellite analysis of maternity and the mating system in the Gulf pipefish *Syngnathus scovelli*, a species with male pregnancy and sex-role reversal. *Mol. Ecol*. **6**, 203‐213. )doi:10.1046/j.1365-294x.1997.00173.x)
83. Jones AG, Walker D, Avise JC. 2001 Genetic evidence for extreme polyandry and extraordinary sex-role reversal in a pipefish. *Proc. R. Soc. Lond. B*. **268**, 2531‐2535. (doi:10.1098/rspb.2001.1841)
84. Rispoli VF, Wilson AB. 2008 Sexual size dimorphism predicts the frequency of multiple mating in the sex-role reversed pipefish *Syngnathus typhle*. *J. Evol. Biol.* **21**, 30–38. (doi:10.1111/j.1420-9101.2007.01470.x)
85. Jones AG, Rosenqvist G, Berglund A, Avise JC. 1999 The genetic mating system of a sex-role-reversed pipefish (*Syngnathus typhle*): a molecular inquiry. *Behav. Ecol. Sociobiol.* **46,**357–365 (doi:10.1007/s002650050630)
86. Katoh R, Munehara H, Kohda M. 2005. Alternative male mating tactics of the substrate brooding cichlid *Telmatochromis temporalis* in Lake Tanganyika. *Zoo. Sci.* **22**, 555–561. (doi:10.2108/zsj.22.555)
87. Sefc KM, Mattersdorfer K, Sturmbauer C, Koblmüller S. 2008 High frequency of multiple paternity in broods of a socially monogamous cichlid fish with biparental nest defence. *Mol. Ecol.* **17**, 2531–2543. (doi:10.1111/j.1365-294X.2008.03763.x)
88. Zimmermann H, Fritzsche K, Henshaw JM, Katongo C, Banda T, Makasa L, Sefc KM, Bose AP. 2019 Nest defense in the face of cuckoldry: evolutionary rather than facultative adaptation to chronic paternity loss. BMC Evol. Biol. 19. (doi:10.1186/s12862-019-1528-7)
89. Paczolt KA, Passow CN, Delclos PJ, Kindsvater HK, Jones AG, Rosenthal GG. 2015 Multiple mating and reproductive skew in parental and introgressed females of the live-bearing fish *Xiphophorus birchmanni*. *J Hered*. **106**, 57‐66. (doi:10.1093/jhered/esu066)
90. Simmons LW, Beveridge M, Evans JP. 2008. Molecular evidence for multiple paternity in a feral population of green swordtails. *J. Hered.* **99**, 610–615. (doi:10.1093/jhered/esn053)
91. Tatarenkov A, Healey CIM, Grether GF, Avise JC. 2008. Pronounced reproductive skew in a natural population of green swordtails, *Xiphophorus helleri*. *Mol. Ecol.* **17**, 4522–4534. (doi:10.1111/j.1365-294X.2008.03936.x)
92. Luo J, Sanetra M, Schartl M, Meyer A. 2005. Strong reproductive skew among males in the multiply mated swordtail *Xiphophorus multilinieatus* (Teleostei). *J. Hered.* **96**, 346–355. (doi:10.1093/jhered/esi042)
93. Smith CC. 2014 Polyandry and paternity in a wild population of the swordtail *Xiphophorus nigrensis.* *Behav. Ecol. Sociobiol.* **68**, 415–424. (doi:10.1007/s00265-013-1655-x)
94. Green ME, Appleyard SA, White W, Tracey S, Ovenden J. 2017 Variability in multiple paternity rates for grey reef sharks (*Carcharhinus amblyrhynchos*) and scalloped hammerheads (*Sphyrna lewini*). *Sci. Rep*. **7**, 1528. (doi:10.1038/s41598-017-01416-w)
95. Pirog A, Jaquemet S, Soria M, Magalon H. 2015 First evidence of multiple paternity in the bull shark (*Carcharhinus leucas*). *Mar. Freshw. Res.* **68**, 195-201. (doi:10.1071/MF15255)
96. Pirog A, Magalon H, Poirout T, Jaquemet S. 2019 Reproductive biology, multiple paternity and polyandry of the bull shark *Carcharhinus leucas*. *J. Fish Biol*. **95**, 1195‐1206. (doi:10.1111/jfb.14118)
97. Bester-van der Merwe AE, Maduna SN, Hull KL, Bell J, Rossouw C, Wintner SP.  2019 Evidence for multiple paternity and confirmation of an Indo-Pacific origin of blacktip shark *Carcharhinus limbatus* occurring in South Africa. *Afr. J. Mar. Sci.* **41**, 281-289. (doi:[10.2989/1814232X.2019.1653991](https://doi.org/10.2989/1814232X.2019.1653991))
98. Rossouw C, Wintner SP, Bester-Van Der Merwe AE. 2016 Assessing multiple paternity in three commercially exploited shark species: *Mustelus mustelus* , *Carcharhinus obscurus* and *Sphyrna lewini*. *J. Fish Biol.* **89**, 1125–1141. (doi:10.1111/jfb.12996)
99. Daly-Engel TS, Grubbs RD, Bowen BD, Toonen RJ. 2007 Frequency of multiple paternity in an unexploited population of sandbar shark (*Carcharhinus plumbeus*). *Can. J. Fish. Aqua. Sci.* **64**, 198–204. (doi:10.1139/f07-005)
100. Portnoy DS, Piercy AN, Musick JA, Burgess GH, Graves JE. 2007 Genetic polyandry and sexual conflict in the sandbar shark, *Carcharhinus plumbeus*, in the western North Atlantic and Gulf of Mexico. *Mol. Ecol.* **16**, 187–97. (doi:10.1111/j.1365-294X.2006.03138.x)
101. Chapman DD, Wintner SP, Abercrombie DL, et al. 2013 The behavioural and genetic mating system of the sand tiger shark, *Carcharias taurus*, an intrauterine cannibal. *Biol. Lett*. **9**, 20130003. (doi:10.1098/rsbl.2013.0003)
102. Holmes BJ, Pope LC, Williams SM, Tibbetts IR, Bennett MB, Ovenden JR. 2018 Lack of multiple paternity in the oceanodromous tiger shark (*Galeocerdo cuvier*). *R. Soc. Open Sci*. **5**, 171385. (doi:10.1098/rsos.171385)
103. Hernández S, Duffy C, Francis MP, Ritchie PA. 2014 Evidence for multiple paternity in the school shark *Galeorhinus galeus* found in New Zealand waters. *J. Fish Biol*. **85**, 1739‐1745. (doi:10.1111/jfb.12490)
104. Gubili C, Duffy CAJ, Cliff G, Wintner S, Shivji MS, Chapman DD, Bruce BD, Martin AP, Sims DW, Jones CS, Noble LR. 2012 Application of molecular genetics for conservation of the great white shark, *Carcharodon carcharias*, L. 1758. In *Global Perspectives on the Biology and Life History of the Great White Shark* (ed. ML Domeier), pp. 357–380. Boca Raton, FL: CRC Press. (DOI: 10.1201/b11532-28)
105. Boomer JJ, Harcourt RG, Francis MP, Walker TI, Braccini JM, Stow AJ. 2013 Frequency of multiple paternity in gummy shark, *Mustelus antarcticus*, and rig, *Mustelus lenticulatus*, and the implications of mate encounter rate, postcopulatory influences, and reproductive mode. *J. Hered.* **60**, 371–9. (doi:0.1093/jhered/est010)
106. Farrell ED, O’Sullivan N, Sacchi C, Mariani S. 2014 Multiple paternity in the starry smooth-hound shark *Mustelus asterias* (Carcharhiniformes: Triakidae). *Biol. J. Linn. Soc.* 111: 119–125. (doi:10.1111/bij.12179)
107. Byrne RJ, Avise JC. 2012 Genetic mating system of the brown smoothhound shark (*Mustelus henlei*), including a literature review of multiple paternity in other elasmobranch species. *Mar. Biol.* **159**, 749–756 (doi:10.1007/s00227-011-1851-z)
108. Chabot CL, Haggin BM. 2014 Frequency of multiple paternity varies between two populations of brown smoothhound shark, *Mustelus henlei*. *Mar. Biol.* **161**, 797–804 (doi:10.1007/s00227-013-2378-2)
109. Marino IAM, Riginella E, Gristina M, Rasotto MB, Zane L, Mazzoldi C. 2015 Multiple paternity and hybridization in two smooth-hound sharks. *Sci. Rep.* **5**, 12919. (doi:10.1038/srep12919)
110. Mourier J, Buray N, Schultz JK, Clua E, Planes S. 2013 Genetic network and breeding patterns of a sicklefin lemon shark (*Negaprion acutidens*) population in the Society Islands, French Polynesia. *PLoS One*. **8**, e73899. (doi:10.1371/journal.pone.0073899)
111. Feldheim KA, Gruber SH, Ashley MV. 2002 The breeding biology of lemon sharks at a tropical nursery lagoon. *Proc. R. Soc. Lond. B*. **269**, 1655–1661. (doi:10.1098/rspb.2002.2051)
112. Feldheim KA, Gruber SH, Ashley MV. 2004 Reconstruction of parental microsatellite genotypes reveals female polyandry and philopatry in the lemon shark, *Negaprion brevirostris*. *Evol.* **58,** 2332–2342. (doi:10.1111/j.0014-3820.2004.tb01607.x)
113. DiBattista JD, Feldheim KA, Gruber SH, Hendry AP. 2008. Are indirect benefits associated with polyandry? Testing predictions in a natural population of lemon sharks. *Mol. Ecol.* **17**, 783–795. (doi:10.1111/j.1365-294X.2007.03623.x)
114. Fitzpatrick SF. 2012 Global population genetic structure of the pelagic blue shark (*Prionace glauca*). PhD Thesis, Queen's University Belfast. (https://ethos.bl.uk/OrderDetails.do?uin=uk.bl.ethos.557408)
115. Chevolot M, Ellis JR, Rijnsdorp AD, Stam WT, Olsen JL. 2007. Multiple paternity analysis in the thornback ray *Raja clavata* L. *J.Hered.* **98**, 712–715. (doi:10.1093/jhered/esm077)
116. Egan AN. 2018 Temporal variation in the frequency of multiple paternity in the Atlantic sharpnose shark (*Rhizoprionodon terraenovae*). MSc Thesis. University of West Florida. (http://etd.fcla.edu/WF/WFE0000606/Egan_Ariel_N_201806_MS.pdf)
117. Griffiths AM, Jacoby DMP, Casane D, McHugh M, Croft DP, Genner MJ, *et al.* 2012 First analysis of multiple paternity in an oviparous shark, the small-spotted catshark (Scyliorhinus canicula L.). *J. Hered.* **103**, 166–173. (doi:10.1093/jhered/esr112)
118. Chapman DD, Prodöhl PA, Gelsleichter J, Manire CA, Shivji MS. 2004 Predominance of genetic monogamy by females in a hammerhead shark, *Sphyrna tiburo*: Implications for shark conservation. *Mol. Ecol.* **13**, 1965–1974. (doi:10.1111/j.1365-294X.2004.02178.x)
119. Lage CR, Petersen CW, Forest D, Barnes D, Kornfield I, Wray C. 2008 Evidence of multiple paternity in spiny dogfish (*Squalus acanthias*) broods based on microsatellite analysis. *J. Fish Biol.* **73**, 2068–2074. (doi:10.1111/j.1095-8649.2008.02065.x)
120. Veríssimo A, Grubbs D, McDowell J, Musick J, Portnoy D. 2011 Frequency of multiple paternity in the spiny dogfish *Squalus acanthias* in the Western North Atlantic. *J. Hered.* **102**, 88–93. (doi:10.1093/jhered/esq084)
121. Daly-Engel TS, Grubbs RD, Feldheim KA, Bowen BA, Toonen RJ. 2010 Is multiple mating beneficial or unavoidable? Low multiple paternity and genetic diversity in the shortspine spurdog *Squalus mitsukurii*. *Mar. Ecol. Prog. Ser.* **403**, 255–267. (doi:10.3354/meps08417)
122. Nosal A, Lewallen E, Burton R. 2013 Multiple paternity in leopard shark (*Triakis semifasciata*) litters sampled from a predominantly female aggregation in La Jolla, California, USA. *J. Exp. Mar. Biol. Ecol*. **446**, 110-114. (doi:10.1016/j.jembe.2013.05.002)
123. Lyons K, Chabot CL, Mull CG, Paterson Holder CN, Lowe CG. 2017 Who's My Daddy? Considerations for the influence of sexual selection on multiple paternity in elasmobranch mating systems. *Ecol Evol*. **7**, 5603‐5612. (doi:10.1002/ece3.3086)
